# 

**Published:** 1897-02

**Authors:** 


					﻿Vol. XVII.
FEBRUARY, 1897.
NO. 2?
JBHE
HOMEOPATHIC pH^lClAfl,
A Monthly Journal of Homoeopathic Materia Medica
and Clinical Medicine.
rt He is a freeman whom truth makes free, and all are slaves beside."
"Seek the Truth: come whence it may, cost what it will."
EDITED AND PUBLISHED BY
WALTER M. JAMES, M.
PHILADELPHIA : V
No. 1231 LOCUST STH^^ET-
LONDON;
ALFRED HEATH & CO., Sole Agents for Great Britain,
114 Ebury Street, Eaton Square.
“Yearly Subscription, $2.SO, invariably in advance. Single numbers, 2S ets.
Entered at the Philadelphia Post-Office as Second-Class Mail Matter.
				

